# IL36 Cooperates With Anti-CTLA-4 mAbs to Facilitate Antitumor Immune Responses

**DOI:** 10.3389/fimmu.2020.00634

**Published:** 2020-04-15

**Authors:** Qiuxia Qu, Zhiwei Zhai, Jieni Xu, Song Li, Cheng Chen, Binfeng Lu

**Affiliations:** ^1^Department of Immunology, University of Pittsburgh School of Medicine, Pittsburgh, PA, United States; ^2^Center for Pharmacogenetics, School of Pharmacy, University of Pittsburgh, Pittsburgh, PA, United States; ^3^UPMC Hillman Cancer Center, Pittsburgh, PA, United States

**Keywords:** CTLA-4, IL36, treg, immunotherapy, mAb

## Abstract

Despite the great impact on long-term survival of some cancer patients, the immune checkpoint blockade (ICB) therapy is limited by its low response rates for most cancers. There is a pressing need for novel combination immunotherapies that overcome the resistance to current ICB therapies. Cytokines play a pivotal role in tumor immunotherapy by helping initiating and driving antitumor immune responses. Here, we demonstrated that, besides conventional CD4^+^ and CD8^+^ T cells, IL36 surprisingly increased the number of tumor-infiltrating regulatory T (Treg) cells *in vivo* and enhanced proliferation of Tregs *in vitro*. Administration of CTLA-4 monoclonal antibodies (mAbs) strongly enhanced IL36-stimulated antitumor activities through depletion of Tregs. In addition, a cancer gene therapy using the IL36-loaded nanoparticles in combination with CTLA-4 mAbs additively reduced lung metastasis of breast tumor cells. We further showed that the combined therapy of CTLA-4 mAbs and IL36 led to an increase in proliferation and IFN-γ production by CD4^+^ and CD8^+^ T cells when compared to single therapy with CTLA-4 mAbs or IL36. Collectively, our findings demonstrated a new combination therapy that could improve the clinical response to ICB immunotherapy for cancer.

## Introduction

Immune checkpoint blockade (ICB) immunotherapy has revolutionized cancer treatment by increasing the overall survival rates of cancer patients. However, clinical response rates are still low for most cancers (1). Higher response rates are achieved when CTLA-4 and PD-1 inhibitors are administered concurrently, demonstrating rational combination therapy will allow more patients to benefit from immunotherapy (2, 3). The antitumor activities of the checkpoint inhibitors are dependent on the number of tumor antigen-specific T cells, which are only abundant in immunogenic tumors. Because inflammatory cytokines play a key role in promoting tumor immunogenicity (4–9), synergistic integration of cytokine- and ICB-based immunotherapy has potential to greatly advance immunotherapy of cancer.

Many recent studies have established a critical role Interleukin 36 (IL36) plays in promoting adaptive and innate immune responses. IL36 consists of IL36α, IL36β, and IL36γ, also known as IL-1F6, IL-1F8, and IL-1F9, respectively, which are members of the IL-1 family of cytokines (10). They share the same receptor complex, which is composed of the IL36 receptor (IL36R) and IL-1RAcP. IL36 can be induced in keratinocytes, bronchial epithelia, brain tissues, and macrophages and is believed to be an “alarmin” in the damaged tissue (10, 11). IL36 exerts its functions directly on multiple cell types including tissue stromal cells, dendritic cells (DCs), CD4^+^ T cells, CD8^+^ T cells, NK cells, and γδ T cells (12–16). Ample evidence supports a crucial role of IL36 cytokines in promoting autoimmunity (17–20). IL36R-deficient mice were protected from imiquimod-induced psoriasiform dermatitis (21). Accumulating evidence supports an important role of IL36γ in driving Th1 immune responses. Pseudomonas, aeroginosa, or TLR3 ligands, induce high levels of IL36γ expression (22, 23) and T-bet is required for the induction of IL36γ in myeloid cells (24). In addition, IL36γ stimulates Th1 differentiation *in vitro* and IL36R is required for protective immune responses to aspergillus and Bacillus Calmette-Guerin infection (14, 25). Recent studies also show that IL36γ promotes antitumor immune responses through enhancing the effector function of type 1 lymphocytes (16, 26–31). All these data have firmly established an important role of IL36 in promoting immune responses. Nonetheless, whether IL36 can participate in immune regulation and enhance the function of immune checkpoint molecules has not been investigated.

Here, we set out to gain a further mechanistic insight of IL36-mediated antitumor immune responses by focusing on its effect on Treg. We first examined whether IL36 promoted Treg proliferation. We also quantified the number of tumoral Treg in IL36-expressing tumors and control tumors. Since one of the antitumor mechanisms of CTLA-4 mAbs is through depletion of tumor infiltrating Treg, we studied the effect of combination therapy of CTLA-4 mAbs and IL36. Our studies further elucidated the cellular mechanisms of IL36-mediated immune responses and also shed light on novel combination immunotherapy of cancer.

## Materials and Methods

### Tumor Cell Culture and Generation of IL36-Expressing Cell Lines

B16 and 4T1.2 cells were cultured in RPMI1640 medium plus 10%FCS. The IL36γ-expression vector was transfected into B16 cells using Lipofectamine 2000 (Invitrogen Life Technologies) according to the manufacturer's instructions to generate B16 stably expressing IL36. Anempty vector (pcDEF3) was transfected into B16 cells as a control.

### Animals

C57BL/6 and BALB/c were purchased from the Jackson Laboratory. All mice were maintained under specific pathogen-free conditions. All mouse experiments were approved by the Institution Animal Care and Use Committee at University of Pittsburgh.

### Synthesis of PEG2k-Fmoc Conjugated With IL36 Plasmid

The construction of IL36 expression plasmid has been described before (12–16). Briefly, the IL-36γ expression construct was generated by fusing the nucleotide sequence encoding the human CD8αsignal sequence to the 5' end of IL-36γ (G13-S164) sequence downstream the elongation factor alpha promoter. The detailed procedure of synthesis of POEG-st-Pmor polymer was described previously (29). Briefly, POEG-st-Pmor micelles were prepared by the dialysis method. 10 mg of polymer was dissolved in 5 mL of DMSO. The solution was lyophilized and resolubilized in 1 mL PBS. For plasmid DNA complexation, polymeric micelles were diluted to different concentrations in water and mixed with plasmid DNA solution to obtain the desired N/P ratios. The mixture was filtered and the filtrate was precipitated by ice-cold ether/ethanol twice. The crude product was dissolved in water and filtered through a 450 nm filter, followed by lyophilization to yield the powder of purified POEG-st-Pmor-IL36 (29). Mice were treated intravenously with IL-36γ plasmid/POEG-st-Pmor micelles every 3 days for four times.

### Mouse Tumor Experiments

B16 cells were injected intradermally into B6 mice, and the size of tumor was monitored every 2–3 days. B16 and IL36-B16 bearing mice were randomized into two treatment cohorts: (i) control IgG or (ii) CTLA4 monoclonal antibodies (mAbs) (clone 9H10, BioXCell). All antibodies were administered at a dose of 200 μg/mouse through intraperitoneal (i.p.) injection twice per week. Mice were euthanized when the tumor volume reached 2,000 mm^3^. The day of euthanasia was used to calculate survival.

To established murine breast tumor lung metastasis model, BALB/c mice were injected *i.v*. with 4T1.2. Treatments with CTLA-4 mAb, POEG-st-Pmor-IL36 nanoparticles or combination were initiated 24 h after tumor cell injection once every 3 days for 4 times *i.v*. On day 15 post tumor cell injection, all mice were sacrificed. Metastatic 4T1.2 tumor nodules were enumerated after the India ink staining procedure, as reported previously. Briefly, India ink solution was injected through the trachea to inflate the lungs, and the lungs were stained for 5 min. The lungs were then removed and placed in Fekete's solution (70% alcohol, 10% formalin, and 5% acetic acid) for destaining. Tumor nodules did not absorb India ink, which resulted in the normal lung tissue staining black and the tumor nodules remaining white. Tumor nodules were counted blindly by two independent investigators (16).

### Analysis of Tumor-Infiltrating Lymphocytes

Tumors were dissected and transferred into RPMI medium. Tumors were disrupted mechanically using scissors, digested with a mixture of 0.3 mg/ml DNase I (Sigma-Aldrich) and 0.25 mg/ml Liberase TL (Roche) in serum-free RPMI medium for 25 min, and dispersed through a 40-mm cell strainer (BD Biosciences). Flow cytometric analysis was performed using a FACS flow cytometer (BD Biosciences). Combinations of the following fluorochrome-conjugated antibody were used for cell surface or intracellular staining to define populations of CD8, and subsets of CD4 T cells: CD45, CD8 (clone 53-6.7), CD4 (clone GK1.5), Foxp3, PD-1, Tim-3, CD69, Ki-67, CTLA-4. For *ex vivo* restimulation, freshly isolated single-cell suspension was cultured in complete RPMI 1,640 medium containing PMA (50 ng/ml) and ionomycin (500 ng/ml) for 3 h before it was analyzed for IFN-γ production by intracellular staining with IFN-γ mAbs (XMG1.2). Multi-colored flow cytometry analyses were performed on LSR II (BD). Data were analyzed with FlowJo software (Tree Star).

### Determination of IL36R Expression by RT-Quantitative-PCR

To determine IL36R expression, single-cell suspensions were made from spleens and lymph nodes of C57BL/6 mice. Naive CD4^+^T (CD44_low_ CD62L_high_), CD8^+^ T, Treg (CD4^+^CD25^+^) cells were purified by fluorescence-activated cell sorting (FACS). Total RNA was extracted using the TrIzol reagent (Invitrogen Life Technologies) according to the manufacturer's protocol. Total RNA was reverse transcribed using SuperScript II Reverse transcriptase (Invitrogen Life Technologies). The mRNA levels for genes of interest were examined by quantitative RT-PCR using SYBR Green PCR Master Mix (Applied Biosystems). Values obtained with the SDS 2.2 (Applied Biosystems) were imported into Microsoft Excel for analyses and gene expression was calculated using the comparative method (2^−δCt^) for relative quantification by normalization to *GAPDH* gene expression.

### Primary Lymphocyte Culture and Stimulation

Total CD4^+^ T cells were washed twice with staining buffer (PBS0.1% BSA), resuspended in staining buffer containing 5 μM CFSE (Molecular Probes), and incubated at 37°C for 15 min. Five volumes of ice-cold culture medium were added to stop labeling, and cells were washed once with culture medium. Cells were then activated with plate-bound anti-mouse CD3 and CD28 antibodies and stimulated with or without IL36 (100 ng/mL), IL-2 (50 U/mL). After 3 days of incubation, the cell division was determined by measuring CFSE fluorescence by flow cytometry.

### Statistical Analysis

Data (mean ± SEM) are representative of independent experiments. We used the two-tailed unpaired Student's *t*-test, Mann-Whitney U test or the log-rank test (survival studies). *P* < 0.05 was considered as being significant.

## Results

### Tumoral Expression of IL36 Increased the Tumor Associated Treg

**Figure 1:**
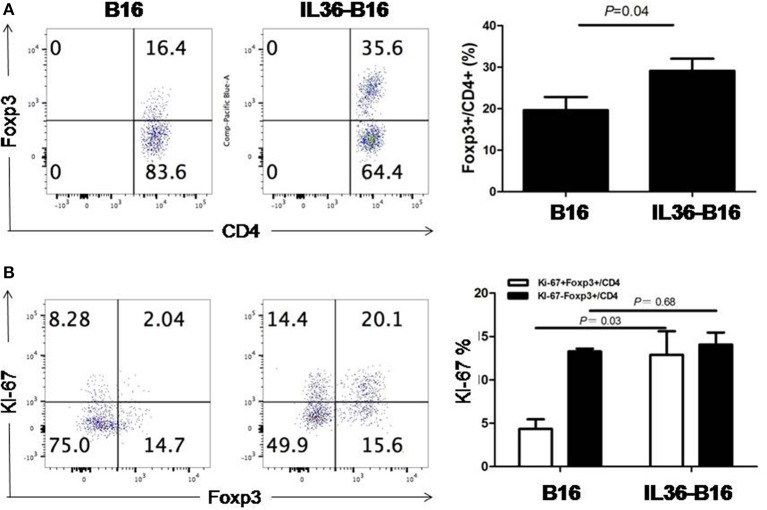
IL-36 increased tumor infiltrated Treg cells and upregulated Ki-67 in Treg cells. A total of 10^4^ B16 or IL-36-B16 cells was injected i.d. into C57BL/6j mice (*n* = 3 mice/group). On day 10–12, tumors were resected and processed to generate single cell suspension. Percentages of Foxp3^+^CD4^+^ T cells **(A)** in tumor infiltrating immune cells and percentages of Ki-67^+^Foxp3^+^ cells **(B)** within the CD4^+^ TIL population were shown. Results are mean ± SEM of three independent experiments.

We have shown that IL36 potently enhanced the effectors function of Th1, CD8^+^ T, NK, and T cells when over-expressed in the tumor tissues. Whether IL36 can exert direct effect on Treg cells is not known. Interestingly, the percentage of tumoral Treg cells was increased greatly in IL36-B16 when compared to B16 tumors (Figure 1A). This was likely due to increases in local proliferation because the percentage of Ki-67^+^Treg cells was greater in IL36-B16 when compared to B16 tumors (Figure 1B). These data indicated that IL36 also induced a self-limiting mechanism through Treg.

### IL36 Promoted the Treg Proliferation *in vitro*

**Figure 2:**
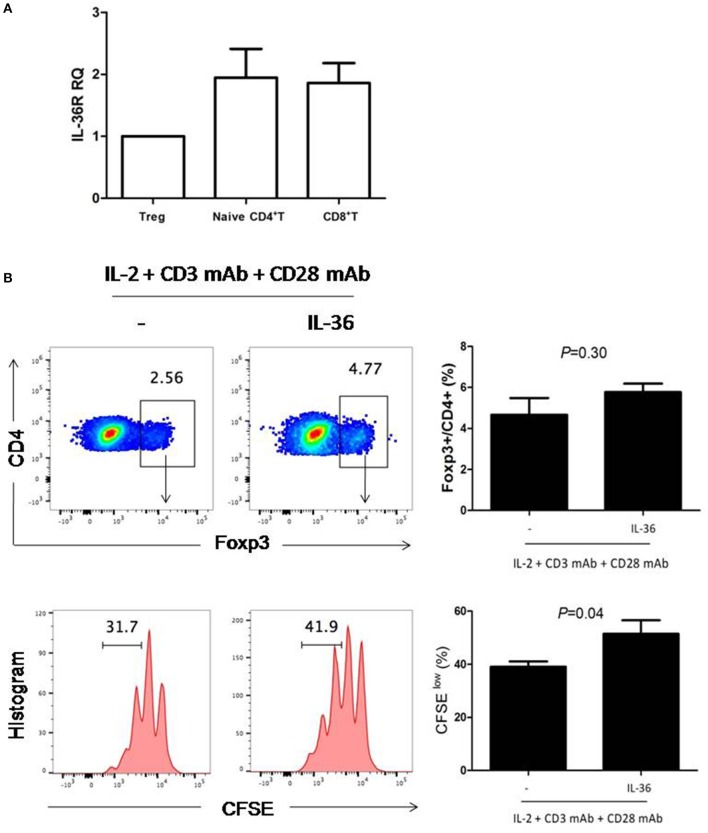
IL-36R was expressed and functional in Treg cells. **(A)**, naïve CD4^+^T cells (Th0 cells), naïve CD8^+^ T cell and Treg were purified by FACS. Total mRNA was isolated for analyses by quantitative RT-PCR. Results represented IL-36R mRNA expression levels relative to *GAPDH*. **(B)**, flow cytometric analysis of Treg cells proliferation responses induced by IL-36-stimulated splenic CD4^+^T cells. Splenic CD4^+^T cells were stimulated with anti-CD3 and anti-CD28 antibodies with or without IL36 for 72 h. CFSE dilution was used to evaluate T cell proliferation responses 72 h following co-culture. Flow cytometry plot is representative of four independent experiments. Results are mean ± SEM of three independent experiments.

In order to further determine whether IL36 can directly increase Treg proliferation, we examined the expression of IL36R in Treg. Similar to results from previous studies, IL36R could be readily detected in both naïve CD4^+^ and CD8^+^T cells (Figure 2A). Interestingly, IL36R was also expressed in Treg (Figure 2A). This was surprising because it was shown that IL36R inhibits generation of the induced Treg (32). We then tried to determine whether IL36 could enhance proliferation of natural Treg *in vitro* with cultured in the presence of CD3 and CD28 mAbs. We found that IL36 indeed promoted Treg proliferation but did not affect the ratio between Treg and conventional CD4^+^ T cells after co-culture (Figure 2B). These data suggest that IL36 could enhance the proliferation of both Treg and conventional T cells. The IL36/Treg axis is likely a natural negative feedback mechanism that limits an overzealous IL36-driven T cell immune response. Such mechanism, although fit for limiting autoimmunity, poses an obstacle for utilization of IL36 in tumor immunotherapy.

### IL36 Combined With CTLA-4 mAbs Additively Eradicated Tumors

**Figure 3:**
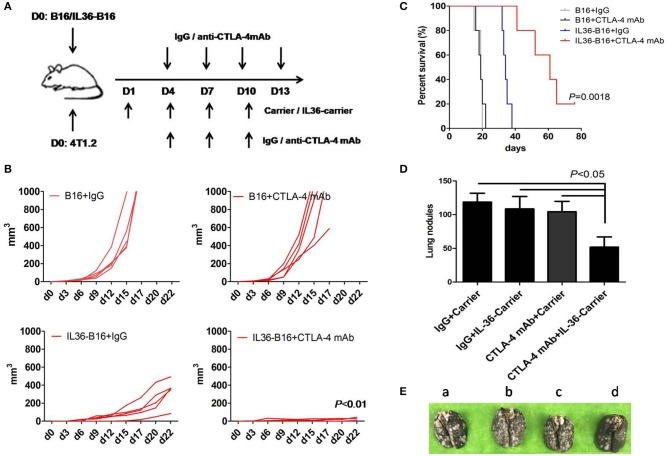
Additively increased antitumor activities with combination of IL36 and CTLA-4 mAbs. **(A)**, Schematic drawing of the experimental setup of IL36 and CTLA-4 mAbs treatment. **(B)**, Individual growth curves of B16 and IL36-B16 tumors with or without CTLA-4 mAbs treatment (*n* = 5 mice/group). **(C)**, Long-term survival of different treatment groups was shown (*n* = 5 mice/group). **(D,E)**, Therapeutic studies were conducted using the 4T1.2 metastatic lung cancer model [*n* = 5 mice/group, **(E)** a, IgG plus Carrier group; b, IgG plus IL36-Carrier group; c, CTLA-4 mAbs plus Carrier group; d, CTLA-4 mAbs plus IL36-Carrier group]. Treatment with CTLA-4 mAbs, PEG2k-Fmoc-IL36 nanoparticles or combination was initiated after tumor cell injection. The presented is representative of samples in one out of three independent experiments. Data (mean ± SEM) are representative of three independent experiments.

One of the antitumor mechanisms of CTLA-4 mAbs is depletion of tumoral Treg (33–35). We therefore hypothesize that combination of tumoral expression of IL36 and CTLA-4 mAbs might additively increase antitumor activities. We administered CTLA-4 mAbs 4 days after subcutaneous implantation of B16 cells or IL36-B16 cells, when tumors were established with an average diameter of 2 mm. We used the B16 melanoma cells because they represent an aggressive murine tumor model and are highly resistant to various immunotherapies. Consistent with previous studies, CTLA-4 mAbs failed to control tumor growth, and tumoral expression of IL36 had pronounced antitumor functions. Combination of CTLA-4 mAbs and IL36 expression produced much greater antitumor efficacy with reduced tumor growth rates and much prolonged survival (Figures 3A–C).

Our previous data show that drug-loaded PEG2k-Fmoc micelles are stable in the blood and are highly effective in selective delivery gene expressing constructs to the lung tumor tissues (29). Our nanocarrier was designed to target both lungs and distant solid tumors (29). Selective accumulation of the nanocarrier is largely attributed to the leaky tumor vasculature. On the other hand, the effective accumulation in the lung is likely due to the interaction of tertiary amine moiety with negatively charged cell membrane in the lung (36). Amine-containing basic compounds have been reported to be predominantly accumulated in the lung due to the specific binding to acidic phospholipids on the cell membrane, which is abundantly distributed in lung tissue. Using this approach, IL36 is locally specifically expressed in tumor cells and lung tissue cells to avoid potential toxicity of *i.v*. injection of a recombinant IL36 protein. We then determine the effectiveness of this gene therapy strategy for delivery of IL36 expression plasmids to tumor and synergy between the IL36 gene therapy and CTLA-4 mAbs. The therapeutic studies were conducted in a 4T1.2 lung metastatic model using a nanomicellar carrier that is based on a prodrug conjugate of PEG, Fmoc with the IL36 plasmid (PEG2k-Fmoc-IL36) (29). We evaluated the lungs of these mice for metastatic nodules and found that numerous tumor nodules were visible on the surface of the lungs of control mice, whereas only mice received both PEG2k-Fmoc-IL36 and CTLA-4 mAbs had significantly fewer lung nodules (Figures 3D,E). These results suggest that local IL36 expression in combination with CTLA-4 mAbs was sufficient to inhibit the growth of metastatic colonies in the lung.

### CTLA-4 mAbs Administration Led to Treg Cell Depletion in Tumor Tissues

**Figure 4:**
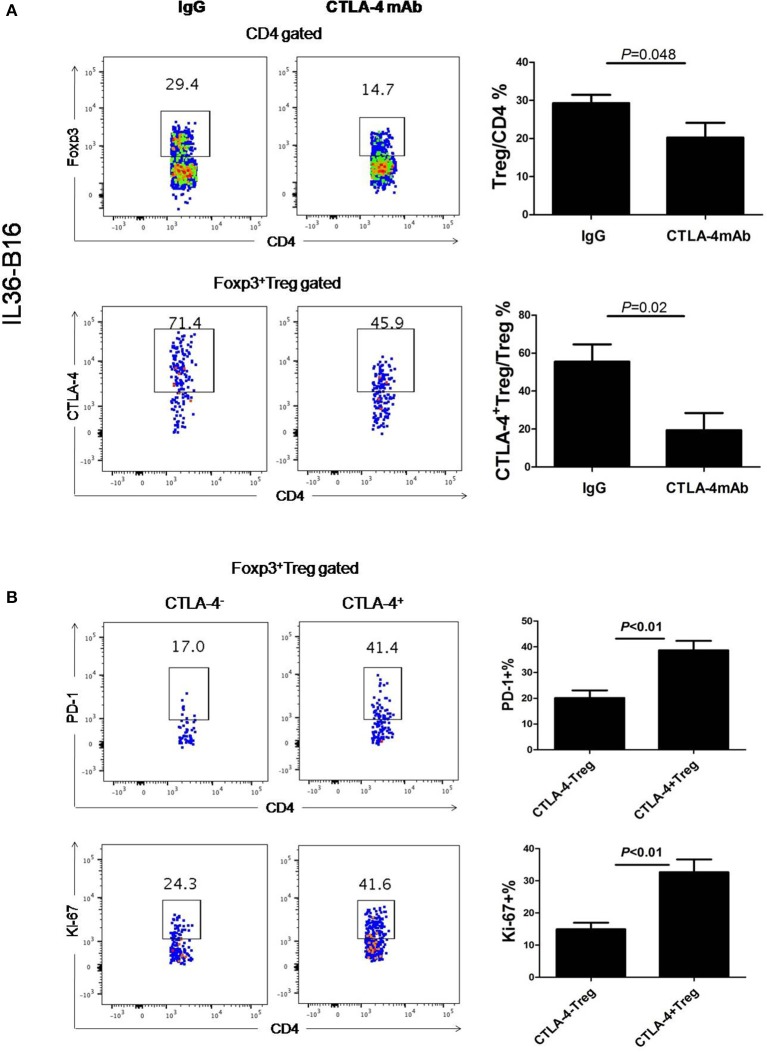
Administration of CTLA-4 mAbs decreased Treg cell population in tumors. **(A)**, Representative flow cytometry plot showing percentages of Foxp3^+^CD4^+^ TIL and CTLA-4^+^Foxp3^+^ TIL in IL-36-B16 tumors after CTLA-4 mAbs treatment (*n* = 3 mice/group). **(B)**, Representative flow cytometry plot showed PD-1 and Ki-67 expression on CTLA-4^+^Foxp3^+^ and CTLA-4^−^Foxp3^+^ T cell subsets in IL-36-B16 tumors. Data (mean ± SEM) are representative of three independent experiments.

Consistent with previously data (37), administration of CTLA-4 mAbs after tumor challenge resulted in a reduced frequency of Foxp3^+^ cells in the B16-IL36 tumor (Figure 4A). This is due to the fact that approximately 70% of the tumoral Treg expressed CTLA-4 (Figure 4A). Interestingly, CTLA-4^+^Treg expressed higher levels of inhibitory molecules PD-1 and Ki-67 than their CTLA-4^−^ counterparts (Figure 4B). These data together suggested thatCTLA-4^+^Treg cells are a more activated Treg subset. Therefore, administration of CTLA-4 mAbs likely resulted in depletion of the activated CTLA-4^+^Treg subset.

### Combination of IL36 and CTLA-4 mAb Resulted in Higher Type 1 Immune Responses in Tumor

**Figure 5:**
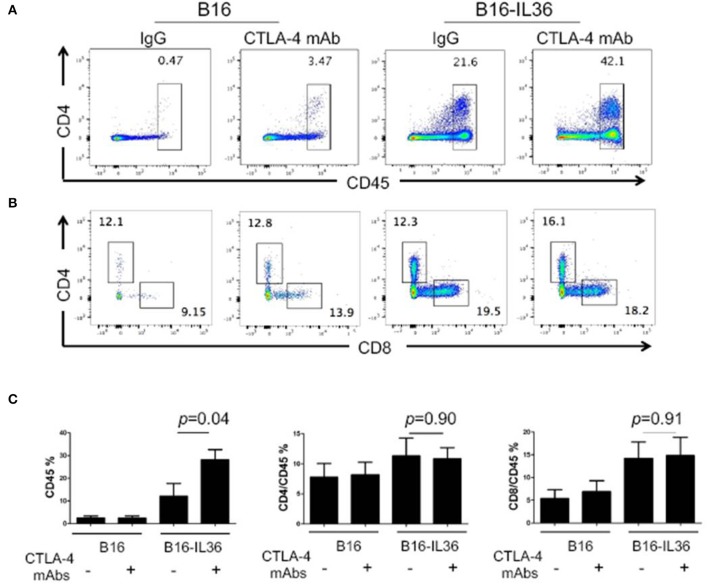
Combination of tumoral expression of IL-36 and anti-CTLA-4 mAbs additively enhanced CD45^+^immune cell infiltration in the tumor microenvironment. C57BL/6 mice were injected i.d. with 10^4^ B16 or B16-IL-36 cells and were subsequently treated with CTLA-4 or control mAbs (*n* = 3 mice/group). Tumors were resected after treatment and subjected to flow cytometry analysis. Representative flow cytometric plots showed CD45^+^ cells **(A,C)**, CD4^+^T and CD8^+^T cells **(B,C)** in tumor. Results are mean ± SEM of 3 independent experiments.

In order to further investigate the protective mechanism, we characterized the immune cells from the tumor tissues by flow cytometry. Compared to control tumors, which were infiltrated with low numbers of immune cells, anti-CTLA-4 mAbs treatment alone did not increase the number of CD45^+^ immune cells (Figure 5). Consistent with our prior report, we found that CD45^+^ immune cells were significantly increased in IL36-B16 tumors when compared to B16 tumors. Combination of IL36 and CTLA-4 mAbs resulted in even greater increases in CD45^+^ immune cells in tumor (Figure 5). Despite increases in the total CD45^+^ tumor infiltrating immune cells, the percentage of CD4^+^ and CD8^+^ T cells were not changed by IL36 or combined administration of IL36 and CTLA-4 mAbs (Figure 5). These data suggest that IL36 and CTLA-4 mAbs additively increase the tumor inflammation.

**Figure 6:**
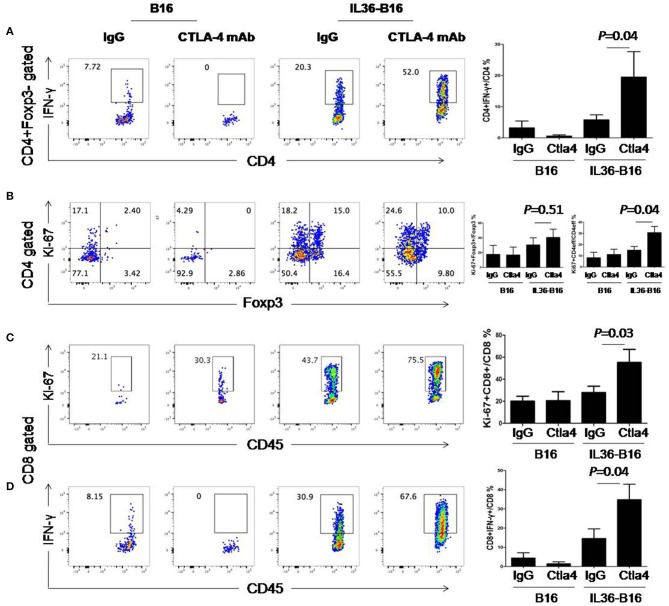
Combination of tumoral expression of IL-36 and anti-CTLA-4 mAbs additively enhanced type 1 immune responses in the tumor microenvironment. C57BL/6 mice were injected i.d. with 10^4^ B16 or B16-IL-36 cells and were subsequently treated with CTLA-4 or control mAbs (*n* = 3 mice/group). Tumors were resected after treatment and subjected to flow cytometry analysis. Representative flow cytometric plots showed IFN-γ^+^Foxp3^−^CD4^+^T cells **(A)**, Ki-67^+^Foxp3^−^CD4^+^T cells **(B)**, Ki-67^+^CD8^+^ T cells **(C)**, IFN-γ^+^CD8^+^ T cells **(D)**. Results are mean ± SEM of three independent experiments.

We then examined whether these treatments resulted in alteration of T cell functions and proliferation. Compared to IL36 or CTLA-4 mAbs alone, combination of the two led to further increase of percentages of IFN-γ^+^ CD4^+^ and CD8^+^ T cells (Figure 6). Likewise, combination of IL36 or CTLA-4 mAbs led to an increase of proliferating CD4^+^ and CD8^+^ T cells (Figure 6). These data indicated that IL36 and CTLA-4mAbs additively increased the effector function and expansion of type 1 T cells in the TME.

## Discussion

In this study, we found that IL36R was expressed in Treg cells. IL36 promoted Treg proliferation *in vitro* and expansion in tumors *in vivo*. In addition, we showed that CTLA-4 mAbs treatment led to a drastic reduction in Treg in IL36-expressing tumors. Furthermore, we demonstrated that IL36 and CTLA-4 mAbs additively promoted antitumor immune responses and greatly prolonged survival of tumor-bearing mice. Collectively, our data indicate that combination of IL36 and CTLA-4 mAbs is a more effective immunotherapy for tumor than individual treatment with IL36 or CTLA-4 mAbs.

Our study has revealed that IL36 regulates a complex cellular network involving both effector and regulatory T cells. We showed previously that IL36R was expressed on both conventional CD4 and CD8 T cells. We and other have shown that IL36 promotes proliferation and IFN-γ of Th1 and CD8 T cell (14, 16). Besides Th1 cells, IL36 has been shown to promote Th9 differentiation. Interestingly, IL36 was shown to inhibit generation of the induced Treg in culture (32). The role of IL36 on natural Treg is not known. In this study, we showed that Treg also expressed IL36R. In addition, IL36 increased Treg proliferation in culture. Moreover, we observed an increase in the percentage and proliferation of Treg in IL36-expressing tumors when compared to control tumors. These data suggest that IL36 induces a self-limiting mechanism mediated by activation of natural Treg cells to contain immune pathology.

It was initially thought the antitumor mechanism of CTLA-4 mAbs was by removing inhibitory signals in the costimulatory pathway (38, 39). CTLA-4 was established as the first negative checkpoint regulatory molecule expressed on activated conventional CD4^+^ and CD8^+^ T cells through a set of experiments using CTLA-4 mAbs (40–43). This concept was further supported by evidence came from analysis of the CTLA-4^−/−^ mice (44–46). CTLA-4^−/−^ mice developed a severe lymphoproliferative disorder and mice die between 18 and 28 days of age. In CTLA-4^−/−^ mice, most of peripheral T cells displayed activated phenotype and secreted effector cytokines and massive lymphocytic infiltration into non-lymphoid tissues are observed. Besides effector T cells, CTLA-4 is also expressed on Treg cells. Ample evidence supports a critical role of CTLA-4 in mediating the function of Treg through downregulating B7/CD28 costimulation (47, 48). Importantly, recent data demonstrated that the antitumor activity of CTLA-4 mAbs seems to be dependent on its Treg-depleting activities (33–35). Therefore, likely both Treg-depletion and reverse of checkpoint inhibition are involved the antitumor function of CTLA-4 mAbs. Our studies support the mechanism of combinatorial effect between IL-36 and CTLA-4 mAbs is at least through Treg. We would expect complete Treg deletion is additive with IL36 treatment and further enhances the antitumor activity of IL36. We have decided to focus on using CTLA4 mAbs in this study due to a clearer pathway for clinical application because there is no other Treg-depletion drug that has been FDA-approved.

Our study suggests that IL-36-based immune therapy of cancer should provide new opportunities for enhancing the immune “checkpoint”-based approach. Our data further demonstrated that depletion of Treg by CTLA-4 mAbs unleashed the power of IL36-mediated tumor immunotherapy. Since CTLA-4 mAbs has been approved for immunotherapy of melanoma and is in clinical trial for combination with PD-1 mAbs (49–51) our data suggest combination of IL36 with CTLA-4 mAbs might be of clinical significance to further increase its efficacy.

## Data Availability Statement

The datasets analyzed in this article are not publicly available. Requests to access the datasets should be directed to binfeng@pitt.edu.

## Ethics Statement

The animal study was reviewed and approved by the Institution Animal Care and Use Committee at University of Pittsburgh.

## Author Contributions

QQ, CC, and BL conceived of the study, participated in its design, coordination, and drafted the manuscript. ZZ, JX, and SL performed 4T1.2 experiments.

### Conflict of Interest

The authors declare that the research was conducted in the absence of any commercial or financial relationships that could be construed as a potential conflict of interest.
